# Dynamic changes of emergency visits: a retrospective observational study

**DOI:** 10.1186/s12873-022-00654-0

**Published:** 2022-06-11

**Authors:** Qihui Liu, Ranran Xin, Yibo Zhao, Muming Yu, Chunjie Jin, Songtao Shou, Yanfen Chai, Heng Jin

**Affiliations:** 1 Department of Emergency Medicine, Tianjin Medical University General Hospital, Tianjin, China; 2 Department of Critical Care Medicine, Tianjin Beichen Hospital, Tianjin, China; 3 Information Department, Tianjin Medical University General Hospital, Tianjin, China

**Keywords:** Epidemiology, Disease spectrum, Respiratory disease, Digestive disease, Circulatory disease, COVID-19

## Abstract

**Background:**

With more emergency visits, there is increasing pressure to provide emergency medical services globally and locally. This study aimed to investigate the epidemiological characteristics and the disease spectrum of patients presenting in the last three years to the Department of Emergency Medicine of Tianjin Medical University General Hospital, a tertiary hospital in Tianjin, China, to improve the services of the emergency medicine department.

**Methods:**

A retrospective study was conducted on all patients in the Department of Emergency Medicine of Tianjin Medical University General Hospital from Jan 1, 2017, 00:00:00 to Dec 31, 2020, 23:59:59, including variables like medical record number, gender, age, date of admission, principal diagnosis. The data were analyzed by SPSS statistical software; statistical charts were prepared by GraphPad Prism9.0 and SPSS 20.0; statistical tables were made by Microsoft Excel.

**Results:**

A total of 1,314,916 patients presented to the Department of Emergency Medicine of Tianjin Medical University General Hospital from Jan 1, 2017, 00:00:00 to Dec 31, 2020, 23:59:59. In terms of gender distribution, the male–female ratio was 0.78∶1. As for age distribution, patients aged 60–69 were the most (23.47%), and patients younger than 20 years were the least (2.80%). Concerning monthly data, the number of visits peaked during January and December. The distribution of daily visits showed the feature of three highs and a low. The top three prevalence diseases in the emergency disease spectrum were respiratory, cardiovascular, and digestive diseases. The respiratory system was the most common in patients with infectious diseases (200,912, accounting for 86.97%). Among the patients suffering from infectious diseases, the number of patients with respiratory infections peaked in 2019 (73,530) and was the lowest in 2020 (20,078).

**Conclusions:**

From 2017 to 2019, the demand for emergency services in Tianjin Medical University General Hospital continued to increase, but it was greatly affected by COVID-19 in 2020. This emergency department is mainly for patients with respiratory system, circulatory system and digestive system diseases, and its treatment time is relatively centralized. The prevention of diseases for people of all ages, especially female patients and the elderly, should be strengthened, and emergency medical resources should be allocated reasonably according to the peak months and crowed periods of patients.

**Supplementary information:**

The online version contains supplementary material available at 10.1186/s12873-022-00654-0.

## Introduction

Apart from thousands of emergency patients, the restricted time of clinician's consolation, and limited medical history information, the critical and complicated conditions of patients often require medical staff in the emergency department to treat them effectively and efficiently. At the same time, the disease spectrum of emergency departments in different regions varies greatly due to genetic, environmental, and economic factors of populations in different areas. Studies have shown that by carrying out epidemiological research, medical staff can not only understand the epidemiological characteristics of population but also provide better emergency medical services to meet the growing needs of patients [1–3]. The Department of Emergency Medicine of Tianjin Medical University General Hospital is one of the first emergency departments established in China. It is now a comprehensive emergency medical center to treat complicated and critical patients in Tianjin. Therefore, we conducted a retrospective study on the relevant data of patients presented to the Department of Emergency Medicine of Tianjin Medical University General Hospital and analyzed the epidemiological characteristics of these patients.

## Materials and methods

### Data source

The Department of Emergency Medicine of Tianjin Medical University is an independent department, and its subordinate departments include internal medicine, surgery, gynecology, ENT, ophthalmology, stomatology, orthopedics, neurology, neurosurgery, and dermatology. The pediatric emergency department, fever clinic, and intestinal emergency department of this hospital are independent departments, so the related patients were excluded in this trial.

The data of patients presented to the Department of Emergency Medicine of Tianjin Medical University General Hospital from Jan 1, 2017, 00:00:00 to Dec 31, 2020, 23:59:59 were collected from the Information Center of Tianjin Medical University General Hospital.

### Key measures

A retrospective study was conducted on all patients in the Department of Emergency Medicine of Tianjin Medical University General Hospital from Jan 1, 2017, 00:00:00 to Dec 31, 2020, 23:59:59, including variables like medical record number, gender, age, date of admission, principal diagnosis. The diagnostic criteria were in accordance with the guidelines of the International Classification of Diseases (ICD-10). Data is collected by the electronic system of the information center of this hospital. The date of admission is the time of the initial clinician's consultation. When there are multiple diagnoses, only the first diagnosis is collected. When patient's diagnosis is not clear, the classification is based on the chief complaint.

Inclusion criteria: The patient's medical records are complete, including medical record number, gender, age, the date of admission, and principal diagnosis.

Exclusion criteria: The patient's medical records are incomplete, missing any of the medical record number, gender, age, the date of admission, and principal diagnosis.

As the data provided by the Information Department is complete, including medical record number, gender, age, visit time, and diagnosis name, no patient in this trial was excluded.

### Statistical analysis

The data were analyzed by SPSS statistical software; statistical charts were prepared by GraphPad Prism9.0 and SPSS 20.0; statistical tables were made by Microsoft Excel. The data were presented as mean ± standard deviation (x ± SD), and enumeration data were expressed as composition ratio.

## Result

### Distribution of patients in different years

From Jan 1, 2017 to Dec 31, 2020, a total of 1,314,916 patients presented to the Department of Emergency Medicine of Tianjin Medical University General Hospital (Table 1).

**Table 1:** The distribution of number, gender and age of patients admitted to the emergency department of Tianjin Medical University General Hospital from 2017 to 2020 (cases, %)

Index	2017	2018	2019	2020
The total	310,758	343,498	404,966	255,694
Gender				
Male	137,904(44.38)	148,616(43.27)	172,598(42.62)	118,888(46.50)
Female	172,851(55.62)	194,882(56.73)	232,368(57.38)	136,806(53.50)
Unknown Gender	3(0.00)	0 (0.00)	0 (0.00)	0 (0.00)
Age				
Mean ± Standard deviation	56.27 ± 19.445	55.55 ± 19.661	54.98 ± 19.851	59.24 ± 18.291
< 20	8,368 (2.69)	10,320 (3.00)	13,919 (3.44)	4,179 (1.63)
20 ~ 29	27,072 (8.71)	31,957 (9.30)	37,733 (9.32)	14,798 (5.79)
30 ~ 39	42,829 (13.78)	51,164 (14.89)	64,858 (16.02)	29,934 (11.71)
40 ~ 49	29,724 (9.56)	31,053 (9.04)	36,410 (8.99)	22,931 (8.97)
50 ~ 59	46,654 (15.01)	50,072 (14.58)	54,465 (13.45)	38,087 (14.90)
60 ~ 69	70,736 (22.76)	78,492 (22.85)	93,374 (23.06)	65,951 (25.79)
70 ~ 79	46,430 (14.94)	49,692 (14.47)	58,702 (14.50)	46,330 (18.12)
≥ 80	38,945 (12.53)	40,748 (11.86)	45,505 (11.24)	33,484 (13.10)

#### Gender and age

Among the 1,314,916 patients presented, the oldest patient was 109 years old. The proportion of patients less than 20 years old was the smallest (36,786, 2.80%), and patients aged 60–69 years accounted for the largest proportion (308,553; 23.47%). The total number of male patients was 578,006 (43.96%), and the total number of female patients was 736,907 (56.04%); therefore, the male–female ratio was 0.78: 1. Also, there were three patients with unknown gender (Table 1).

### Distribution of visit time

**Fig. 1:**
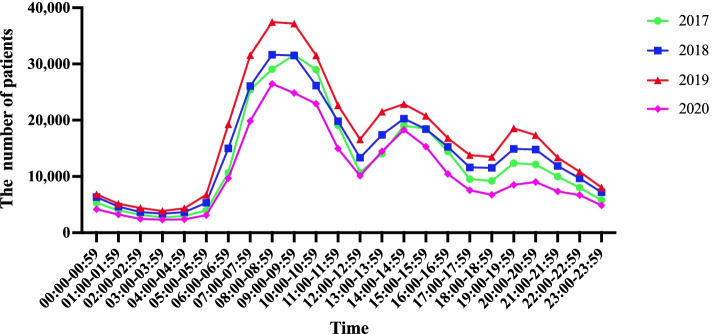
The time distribution of patients admitted to the emergency department of Tianjin Medical University General Hospital from 2017 to 2020

The daily peak period was observed during three time slots, i.e., 08:00–11:00, 14:00–16:00, and 19:00–22:00, whereas the least number of visits occurred during the time slot of 01:00–05:00 (Fig. 1).

### Distribution of weekly visits

**Fig. 2:**
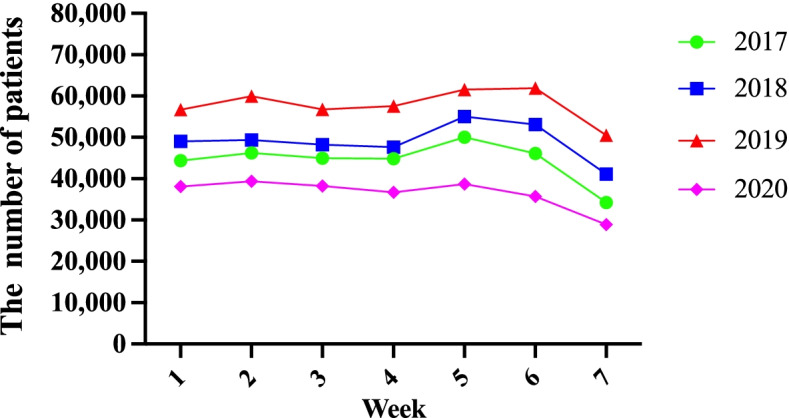
The weekly distribution of patients admitted to the emergency department of Tianjin Medical University General Hospital from 2017 to 2020

Analysis of the weekly distribution of the admitted patients revealed that in 2017, 2018, and 2020, the peak period of patients in the emergency department appeared on Friday, while the off-peak period appeared on Sunday; in 2019, the peak period was observed on Saturday, and the reverse was observed on Sunday (Fig. 2).

### Distribution of monthly visits

**Fig. 3:**
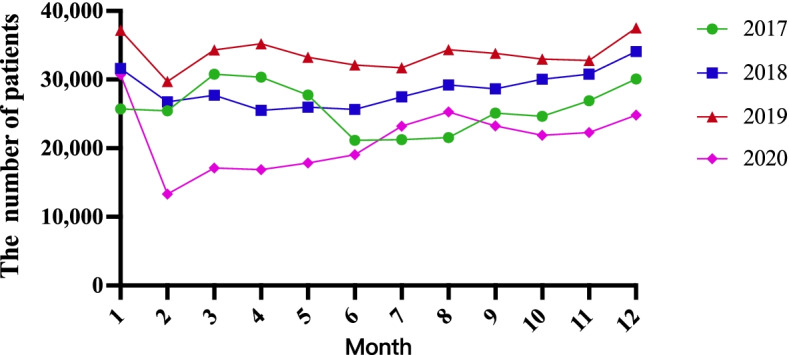
The monthly distribution of patients admitted to the emergency department of Tianjin Medical University General Hospital from 2017 to 2020

Analysis of the monthly distribution of the admitted patients revealed that in 2017, the peak period of patients in the emergency department appeared in March, while the off-peak period appeared in June; in 2018, the peak period was observed in December, and the reverse was observed in April; in 2019, the peak period appeared in December, whereas the off-peak period appeared in February; in 2020, the number of patients peaked in January, and the least number of patients were admitted in February (Fig. 3).

### Distribution of patients among different departments

Among the patients presented to the Department of Emergency Medicine of Tianjin Medical University General Hospital from Jan 1, 2017, to Dec 31, 2020, patients with internal medicine-related diseases were the most, accounting for more than 63% of the cases, followed by surgery (2%–6%) (Table 2).

**Table 2:** The disease spectrum of patients admitted to the emergency department of Tianjin Medical University General Hospital from 2017 to 2020 (cases, %)

The disease spectrum		2017		2018		2019		2020
Internal Medicine	209,343 (67.37)		228,249 (66.45)		263,640 (65.10)		161,304 (63.08)	
Respiratory system	64,077 (20.62)		79,143 (23.04)		96,902 (23.93)		31,880 (12.47)	
Cardiovascular system	61,260 (19.71)		60,189 (17.52)		59,151 (14.61)		46,807 (18.31)	
Digestive system	25,335 (8.15)		29,172 (8.49)		43,983 (10.86)		26,685 (10.44)	
Urinary system	10,858 (3.49)		13,497 (3.93)		14,270 (3.52)		9,051 (3.54)	
Metabolism Endocrinology	29,766 (9.58)		27,977 (8.14)		30,177 (7.45)		25,993 (10.17)	
Rheumatic immune system	4,609 (1.48)		2,589 (0.75)		2,184 (0.54)		1,692 (0.66)	
Blood system	10,731 (3.45)		13,003 (3.79)		14,027 (3.46)		15,912 (6.22)	
Nervous system	2,172 (0.70)		2,120 (0.62)		2,244 (0.55)		2,803 (1.10)	
Poisoning	535 (0.17)		559 (0.16)		702 (0.17)		481 (0.19)	
Surgery	15,083 (4.85)		14,344 (4.18)		11,853 (2.93)		14,554 (5.69)	
General Surgery	9,169 (2.95)		9,118 (2.65)		6,355 (1.57)		11,279 (4.41)	
Orthopedics	921 (0.30)		899 (0.26)		1,100 (0.27)		1,106 (0.43)	
Neurosurgery	4,993 (1.61)		4,327 (1.26)		4,398 (1.09)		2,169 (0.85)	
Obstetrics and Gynecology	983 (0.32)		1,342 (0.39)		1,072 (0.26)		602 (0.24)	
Dermatology	1,871 (0.60)		1,171 (0.34)		1,461 (0.36)		915 (0.36)	
Otorhinolaryngology	3,118 (1.00)		4,470 (1.30)		5,235 (1.29)		2,141 (0.84)	
Stomatology	282 (0.09)		326 (0.09)		391 (0.10)		397 (0.16)	
Ophthalmology	890 (0.29)		566 (0.16)		552 (0.14)		565 (0.22)	
Psychiatry	928 (0.30)		443 (0.13)		310 (0.08)		382 (0.15)	
Other	78,260 (25.18)		92,587 (26.95)		120,452 (29.74)		74,834 (29.27)	
The total	310,758		343,498		404,966		255,694	

### Disease spectrum

In 2017, 2018, and 2019, respiratory diseases were most reported; however, in 2020, cardiovascular diseases were the most (Tables 2 and 3).

**Table 3:** The distribution of the top three diseases of each common system in the disease spectrum of patients admitted to the emergency department of Tianjin Medical University General Hospital from 2017 to 2020 (cases, %)

	2017		2018		2019		2020	
	Disease	Number of patients	Disease	Number of patients	Disease	Number of patients	Disease	Number of patients
Respiratory system	Respiratory tract infection	24,915	Respiratory tract infection	28,159	Respiratory tract infection	34,442	Respiratory tract infection	9,104
	Pneumonia	8,455	Pneumonia	11,518	Bronchitis	15,971	Pneumonia	4,980
	Bronchitis	6,871	Bronchitis	11,374	Pneumonia	14,594	Bronchitis	3,077
Cardiovascular system	Hypertension	22,969	Coronary Heart Disease	20,029	Coronary Heart Disease	18,667	Hypertension	15,411
	Coronary Heart Disease	21,490	Hypertension	19,697	Hypertension	18,441	Coronary Heart Disease	12,171
	Palpitation	3,889	Palpitation	4,993	Palpitation	5,915	Palpitation	5,028
Digestive system	Gastritis	10,980	Gastritis	12,774	Gastritis	17,488	Gastritis	11,304
	Liver injury	3,940	Diarrhea	4,230	Diarrhea	4,231	Gastric mucosal lesions	2,826
	Diarrhea	3,362	Liver injury	2,317	Gastric mucosal lesions	3,948	Liver injury	1,504

### Distribution of infections among the common systems of patients

Among the patients presented to the Department of Emergency Medicine of Tianjin Medical University General Hospital from Jan 1, 2017, to Dec 31, 2020, 23,1020 patients reported symptoms of infection. Among them, patients with symptoms of respiratory infection were the most (200,912; 86.97%), followed by patients with symptoms of urinary infection (23,258; 10.07%), and digestive infection (5,160; 2.23%). Among the patients with symptoms of respiratory infection, the number of patients was the highest (73,530) in 2019, and the number of patients was the lowest (20,078) in 2020 (Table 4).

**Table 4:** The distribution of infected patients admitted to the emergency department of Tianjin Medical University General Hospital from 2017 to 2020 (cases, %)

Index	2017	2018	2019	2020
Respiratory infection	48,702 (86.46)	58,602 (87.24)	73,530 (88.42)	20,078 (82.40)
Cardiovascular infection	203 (0.36)	229 (0.34)	253 (0.30)	74 (0.30)
Digestive infection	1,902 (3.38)	1,211 (1.80)	1,477 (1.78)	570 (2.34)
Urinary infection	5,329 (9.46)	6,814 (10.14)	7,673 (9.23)	3,442 (14.13)
Skin and soft tissue infection	190 (0.34)	314 (0.47)	225 (0.27)	202 (0.83)
The total	56,326	67,170	83,158	24,366

## Discussion

### In recent years, patients presenting to the emergency department have continued to increase, but the COVID-19 pandemic affected them dramatically

From 2017 to 2019, the number of patients presented to the emergency department of Tianjin Medical University General Hospital increased every year, with the average annual number of visits exceeding 300,000. At present, the number of patients presenting to the emergency departments of various hospitals in China is increasing, resulting in a shortage of hospital beds. This is supported by the findings of Ramanathan S and Atiq H that reported a steady increase in the number of patients presented to the emergency departments of various hospitals in other countries [4, 5]. The reasons behind this increase may be: (1) The aging of the population and increased health awareness among people in general. Also, the rapid industrialization and the increase in trauma patients have led to a significant increase in overall medical demand. (2) Due to the lack of primary health care facilities and uneven distribution of medical resources, patients prefer to go to large-scale general hospitals with strong comprehensive strength. (3) The implementation of triage diagnosis and treatment is often incomplete, and patients have a long waiting time for outpatient services, so they turn to the emergency department for treatment. (4) There is no significant difference between the expenses of emergency treatment and outpatient treatment, and the advantage of convenience in the emergency department is more obvious.

However, in 2020, the number of patients presented to the emergency departments of various hospitals has decreased significantly, indicating that the pandemic has changed people's choices of medical and healthcare services. Since the COVID-19 was declared a pandemic by the World Health Organization (WHO) on Mar 11, 2020, it has spread rapidly across the world, forcing people to reduce social activities and endorse self-isolation and telecommuting. Compared to previous years, the reduction in the movement and gathering of people has led to a significant decrease in the number of other types of infections. Meanwhile, the incidence of injuries caused by alcohol consumption, mass riots, and car accidents has significantly reduced. Also, due to the atmosphere of fear created by COVID-19, people have reduced seeking unnecessary medical services. Reports confirm that regular outpatients and patients with sudden illnesses have also shown signs of giving up treatment. According to a report by the Spanish health system [6], during COVID-19 (Mar 16, 2020, to Mar 22, 2020), the number of patients treated for ST-elevation myocardial infarction decreased by 40%. Another study reported that in the early stage of the pandemic in the United States, the number of visits to the emergency department was significantly lower than that in the same period in 2019 [7].

### Most of patients in the emergency department are elderly and young, and the proportion of female patients is large

Among the 1,314,916 patients, 578,006 were males (43.96%), 736,907 females (56.04%), and three were people with unknown gender. The possible reason for the higher number of female patients is that the average life expectancy of women is greater than that of men, and women are more aware of health care than men. This is consistent with the related reports of Satia I [8] and Rozario SS [9].

The age groups of patients in the emergency department were mainly 60–69 years and 30–39 years. With age, the immune system declines, leading to various diseases. Besides, the probability of various accidents is higher in the elderly compared to younger people. In contrast, the younger generations are energetic and have more social interactions in their daily lives, leading to the high incidence of emergency department visits among young people. This result is similar to the report of Shaban [10], while it is inconsistent with the report of Rizoli [11], which states that the number of visits to the emergency department is positively correlated with the age of patients. These differences in findings could be attributed to the fact that the characteristics of the survey sample may be inconsistent with the overall characteristics of the region. Also, the age composition of people or the customs and habits of residents in different regions may vary significantly.

### The visit time of patients in the emergency department is concentrated to certain time periods, and the visit months are concentrated in January and December

According to the statistics of patients in the emergency department from 2017 to 2020, the distribution of their visit time presents the characteristics of "three highs and one low" periods, and the peak period is mainly concentrated at 08:00–11:00, 14:00–16:00, and 19:00–22: 00, whereas the minimum number of visits is mainly concentrated in 01:00–05: 00, which is consistent with the report of Alexander Becker. The following reasons may be behind the occurrence of the peak periods during the day: (1) There are a substantial number of outpatients in various departments, so many patients chose emergency departments to reduce the waiting time; and (2) The elderly patients often choose emergency departments for follow-up visits and medicines. Reasons behind the peak period at night may be the following: (1) The characteristics of respiratory, cardiovascular, and cerebrovascular diseases in elderly patients; (2) Public officials only have time for medical treatment at night; and (3) The increase in collective activities leads to increased incentives for trauma.

In the statistics of patients in the emergency department from 2017 to 2020, the number of emergency visits on weekdays did not vary much, and there was a slight reduction on weekends decreased slightly. Based on the analysis of weekly visits, emergency patients were the most on Friday and the least on Sunday. It is just a normal fluctuation, which is in line with the living habits of people. People tend to seek medical treatment on weekdays and rest on weekends.

In the statistics of patients in the emergency department from 2017 to 2020, the peak of visits occurred during January and December. The reasons may be (1) the lower temperatures, the reduced activity of the population but increased density, and the poor indoor ventilation in winter, which increases the risk of respiratory diseases. (2) The Spring Festival in China occurs close to January because of which the mobility of the population increases significantly, leading to the spread and prevalence of various infectious diseases. (3) Inhalation of cold air can cause vasoconstriction and the contraction of airway smooth muscle, leading to the reduced blood supply to the respiratory tract and impaired movement of respiratory tract cilia. These make it easier for pathogens to accumulate in the respiratory tract and eventually cause the increased incidence of various diseases in the respiratory system [12]. (4) Studies have shown that the stimulation of cold air increases sympathetic nerve activity, resulting in increased catecholamines in the body, leading to vasoconstriction, increased blood pressure, and coronary artery spasm; this leads to an increased burden on the heart and the incidence of cardiovascular disease [13]. (5) Sedentary lifestyle and reduced outdoor activities in winter may be triggers for patients with myocardial infarction [14]. And (6) There are higher seasonal blood pressure changes in winter, which may also increase the risk of intracerebral hemorrhage [15, 16].

### Distribution of disease spectrum in the emergency department

From 2017 to 2020, the top three reported diseases of patients in the emergency department were respiratory, cardiovascular, and digestive diseases. In 2017, 2018, and 2019, respiratory diseases were the most common in the disease spectrum, whereas in 2020, cardiovascular diseases had the highest occurrence rate. This observation is consistent with the report of Alexander Becker [2]. During the pandemic in 2020, the number of patients with respiratory and circulatory diseases increased significantly in the emergency departments of various hospitals in the United States [17].

### Respiratory system

From 2017 to 2020, most of the patients with respiratory diseases were confirmed to suffer from respiratory tract infections. In 2020, because of COVID-19, the number of patients with respiratory diseases decreased, which is related to cutting off the transmission route of respiratory diseases, such as mandatory wearing masks and reducing unnecessary gathering activities. Respiratory diseases are the most commonly reported ailment in emergency medical services, and their mortality rate is the highest. According to the WHO, acute respiratory infection is one of the main causes of death in developing countries [18]. Respiratory tract infection accounts for about 3% of the total patients and 30% of the infected patients, and its prevalence in winter is as high as 6% [19], which indicates an obvious seasonal trend [20]. The Chien-Cheng Jung report states that the release of inflammatory mediators in winter, such as interleukin-1, or tumor necrosis factor-alpha, increased due to changes in temperature [21], which reduced the permeability of the vascular endothelium and inhibited fibrinolysis, resulting in acute respiratory distress syndrome [22]. Besides, the stress response of the respiratory tract to the inhaled cold air increases the concentration of fibrinogen in plasma [23]. Smog in winter, sandstorms, catkins, and pollen in spring pose a serious threat to residents' health. It has been reported that air pollutants can oxidize mitochondria to cause apoptosis or necrosis of macrophages and respiratory epithelial cells, which ultimately leads to a decline in the host's defense against respiratory tract infections or an increase in respiratory responsiveness [24]. Given the peak period of respiratory diseases in winter, the managers of emergency departments should increase the medical staff on duty and carry out proper disinfection of the hospitals. Residents should avoid going to public places as much as possible and adopt personal protection measures, such as wearing masks and maintaining indoor air circulation. For people with poor immunity, such as the elderly and children, influenza vaccination can be given in advance every year.

### Circulatory system

From 2017 to 2020, most patients with circulatory diseases were reported to suffer from hypertension and coronary heart disease. The aging of the population has increased the incidence of critical elderly patients. Chun Shing Kwok [25] reported that cardiovascular disease is the leading cause of death in emergency departments. Lunsky y [26] reported that the proportion of elderly patients due to cardiovascular diseases is relatively large, and the diseases reported by them include myocardial infarction, heart failure, arrhythmia, angina pectoris, and hypertensive emergencies.

Patients with cardiovascular diseases in the emergency department accounted for 14.61–19.71% of the total number of visits. Early health publicity and education for cardiovascular diseases should be the goal of community intervention, such as seeking emergency medical services quickly, public education on early cardiopulmonary resuscitation, equipping defibrillators in public places, and training the public on the correct use of automatic external defibrillators [27]. The treatment of cardiovascular diseases emphasizes rapidness. Therefore, for common and frequently-occurring cardiovascular diseases, medical staff can conduct risk assessments on patients to identify high-risk patients in time and refer them to relevant departments in time.

### Digestive system

During the period of 2017 to 2020, most patients with digestive diseases were reported to suffer from gastritis, liver injury, and diarrhea. Gastritis is a relatively common disease of the digestive system; however, patients with gastritis do not present with typical clinical manifestations [28]. Patients with acute gastritis suddenly have upper abdominal pain, nausea, and vomiting, with nil or minimal symptoms of dyspepsia. Once a patient has obvious symptoms, the patient's condition is often very serious. Therefore, medical staff should provide timely treatment for gastrointestinal bleeding and acute abdominal pain caused by gastritis. Also, they need to equip patients with the knowledge of gastritis-related health care.

### Rational allocate the resources of the emergency medicine department and improve emergency medical services

In this study, it was observed that female patients account for a large proportion of patients in the emergency department. On the one hand, we can continue to study the sex ratio of the population in Tianjin to analyze whether it is a key factor affecting the sex ratio of emergency medicine patients. On the other hand, strengthen the publicity of knowledge on prevention and treatment of common diseases among women, and enhance women's self-hygiene consciousness. At the same time, community medical institutions can carry out screening and follow-up of common female diseases to complete the connection of screening, diagnosis, and treatment.

The study found that emergency patients are mainly elderly and young. Therefore, health care publicity must be focused on the elderly and young patients to raise the health awareness of these two groups. In terms of the distribution of daily and monthly visits, managers of emergency department should try to tilt the allocation of medical staff toward peak periods. Also, during peak periods, vacations or meetings of medical staff should be reduced, so as to ensure a sufficient number of medical staff on duty to improve the efficiency of patients' treatment.

Aiming at the top three diseases in the disease spectrum, measures, such as publicizing the health care knowledge of related diseases, strengthening the guidance of people's diet and health, and improving people's health awareness, should be adopted to avoid the risk factors of related diseases. Also, hospitals should regularly conduct relevant clinical training for medical staff to improve emergency medical services.

### Limitations of the study

First, the sample size of this study is relatively small. Except for the relevant data of patients presented to the emergency department of Tianjin Medical University General Hospital, the relevant data of patients in the emergency departments of other hospitals are not included in this study. Therefore, this study is not comprehensive enough and may not accurately reflect the actual situation of the region or the whole country. Second, the period of the study is not enough to compare the changes of the disease spectrum over a longer period. Finally, this study did not track the prognosis of the patients. Therefore, it is suggested to expand the retrospective study of patient data and analyze patient information more systematically and comprehensively in future studies.

## Conclusions

From 2017 to 2019, the demand for emergency services in Tianjin Medical University General Hospital continued to increase, but it was greatly affected by COVID-19 in 2020. This emergency department is mainly for patients with respiratory system, circulatory system and digestive system diseases, and its treatment time is relatively centralized. The prevention of diseases for people of all ages, especially female patients and the elderly, should be strengthened, and emergency medical resources should be allocated reasonably according to the peak months and crowed periods of patients.

## Supplementary information

**Additional file 1.**

**Additional file 2.**

**Additional file 3.**

**Additional file 4.**

## Data Availability

All materials are owned by the authors and/or no permissions are required. If you need more details about the data, please contact Qihui Liu(email: Liuqihui@tmu.edu.cn).
